# Effects of inflammatory endotypes on disease trajectory in chronic rhinosinusitis with nasal polyps

**DOI:** 10.1016/j.jaci.2025.03.029

**Published:** 2025-04-10

**Authors:** Christina Dorismond, Yash Trivedi, Mason R. Krysinski, Rory J. Lubner, Li-Ching Huang, Sandeep Goswami, Quanhu Sheng, Rakesh K. Chandra, Naweed I. Chowdhury, Justin H. Turner

**Affiliations:** a Department of Otolaryngology-Head and Neck Surgery, Vanderbilt University Medical Center, Nashville, Tenn; b Vanderbilt University School of Medicine, Nashville, Tenn; c Department of Biostatistics, Vanderbilt University Medical Center, Nashville, Tenn; d Department of Otolaryngology-Head and Neck Surgery, University of Alabama–Birmingham, Birmingham, Ala.

**Keywords:** Chronic rhinosinusitis, nasal polyp, cytokine, cluster, endotype, outcomes, polyp recurrence, biologics, dupilumab, principal-component analysis, hierarchical cluster analysis

## Abstract

**Background:**

Although phenotypic features have traditionally guided treatment in chronic rhinosinusitis, recent research has favored categorization on the basis of inflammatory endotype. However, the impact of endotypic differeces on clinical outcomes remains largely unknown.

**Objective:**

We sought to compare disease trajectory, primarily time-to-polyp recurrence, between chronic rhinosinusitis with nasal polyp (CRSwNP) endotypes.

**Methods:**

Samples were obtained from patients with CRSwNP undergoing surgery between 2015 and 2023, and cytokine levels were measured using a multiplex bead assay. Principal-component analysis followed by hierarchical cluster analysis was used to identify endotype clusters. Clinical outcomes were subsequently compared between clusters.

**Results:**

Six CRSwNP disease clusters were identified among the 269 included patients. Cluster 1 (46.5%) was characterized by relatively low inflammation. Cluster 4 (13.3%) and cluster 6 (7.1%) also exhibited low inflammation but with elevated levels of IL-12/IL-21 and CCL5, respectively. Cluster 2 (4.5%) represented a mixed type 1/3 inflammatory endotype (IFN-γ^High^/IL-4^High^/IL-17A^High^), and cluster 3 (10.0%) was characterized by an innate, proinflammatory response (IL-1β^High^/IL-6^High^/IL-8^High^). Cluster 5 (18.9%) exhibited type 2–dominant inflammation (IL-5^High^/IL-9^High^/IL-13^High^). When comparing disease trajectory, cluster 2 (IFN-γ^High^/IL-4^High^/IL-17A^High^) and cluster 4 (IL-12^High^/IL-21^High^) had the shortest time-to-polyp recurrence, whereas cluster 3 (IL-1β^High^/IL-6^High^/IL-8^High^) demonstrated the longest time-to-recurrence (*P* < .001). Time-to-oral steroid course (*P* = .13) and time-to-biologic therapy (*P* = .43) were similar across clusters.

**Conclusions:**

The study highlights the heterogeneous nature of CRSwNP and differences in disease trajectory between endotypes, notably that patients with mixed type 1 and type 3 inflammation demonstrate more recalcitrant disease. These findings suggest that therapies beyond traditional type 2 inflammation treatments may be needed to effectively reduce CRSwNP disease recurrence.

## Graphical Abstract

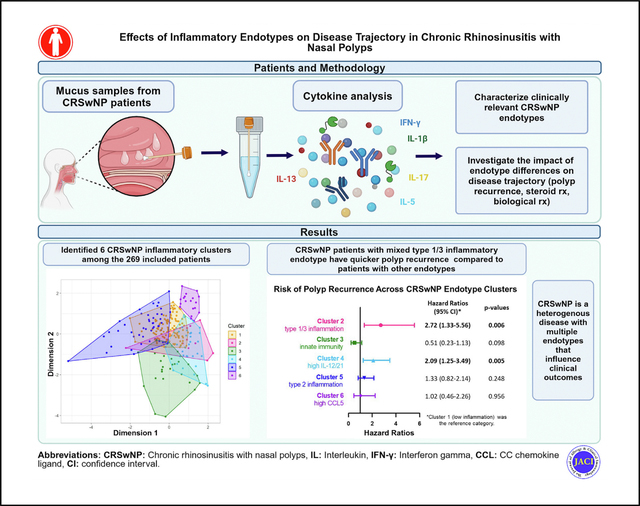

**Capsule summary:** Chronic rhinosinusitis with nasal polyps (CRSwNP) is a heterogeneous disease with multiple endotypes, and CRSwNP patients with a mixed type 1 and type 3 inflammatory endotype have more rapid polyp recurrence and worse disease outcomes compared with patients with a type 2–dominant inflammatory endotype. Although further studies are needed, these findings suggest that tailored therapeutic strategies beyond those targeting traditional type 2 inflammation may be needed for the treatment of severe CRSwNP.

Chronic rhinosinusitis (CRS) with nasal polyps (CRSwNP) is an inflammatory disorder of the paranasal sinuses that results from a complex interplay between genetic predisposition, environmental factors, and dysregulated immune responses.^1–3^ It has traditionally been treated with combined medical and surgical management. More recently, humanized mAbs that target type 2 inflammation, such as dupilumab, omalizumab, and mepolizumab, have emerged as promising treatment modalities for patients with CRSwNP who have failed maximal medical and surgical treatment.^4^

Although treatment decisions for patients with CRSwNP have historically been guided by phenotypic differences, chiefly the presence or absence of nasal polyps, recent studies suggest that significant pathophysiologic diversity exists among patients with this diagnosis. Several translational research studies, including some from our own research group, have characterized distinct and clinically relevant inflammatory CRS endotypes.^2,3,5–15^ However, few have examined how disease trajectory and clinical outcomes vary between CRSwNP endotypes.

Moreover, the clinical course of patients with CRSwNP has been notoriously difficult to predict, with some individuals experiencing rapid polyp recurrence necessitating early repeat surgical or medical interventions and others exhibiting a more stable course.^16,17^ Given that disease recurrence has a substantial impact on patients, manifesting as both a personal burden and an economic strain, understanding the factors influencing clinical trajectory is essential.^18^

Our study aimed to characterize endotypic variation in patients with CRSwNP on the basis of inflammatory biomarkers and explore how this endotype heterogeneity influences disease trajectory, particularly time-to-polyp recurrence.

## METHODS

### Patient selection

After approval by the Vanderbilt University Medical Center Institutional Review Board (IRB no. 121467), patients who presented to rhinology clinics at the Vanderbilt Asthma, Sinus, and Allergy Program and the Vanderbilt Bill Wilkerson Center with bilateral CRSwNP and who underwent endoscopic sinus surgery (ESS) between 2015 and 2023 were selected for inclusion. CRS was diagnosed according to the European Position Paper on Rhinosinusitis and Nasal Polyps and the International Consensus Statement on Allergy and Rhinology.^19–22^ CRSwNP was diagnosed on visualization of nasal polyps with nasal endoscopy in clinic or during surgery. Patients were excluded if they had CRS without nasal polyps (CRSsNP), odontogenic rhinosinusitis, suspected mycetoma, cystic fibrosis, known autoimmune or granulomatous disorder, systemic steroid use within 1 month of surgery, or biologic use within 6 months of surgery. However, patients who were prescribed biologics before surgery but had not yet initiated treatment at time of surgery were included in the study. Moreover, several previous studies have compared patients with CRSwNP and healthy control patients with respect to differences in inflammatory mediators and endotypic characteristics.^2,3,6,8,23^ Therefore, control patients were not included in this study because we aimed to focus on identifying endotypic variability in a phenotypically similar patient population, namely, CRSwNP.

### Mucus collection and cytokine measurement

The method of mucus collection and cytokine quantification has been previously described by our group.^12,14,15,23,24^ In brief, mucus samples were obtained for each patient by placing an absorbent sponge in the middle meatus of the nasal cavity under endoscopic guidance at the beginning of the surgical procedure. Cytokine levels in each sample were measured using a multiplex flow-cytometric bead assay (BD Biosciences, Franklin Lakes, NJ). These included levels of IL-1β, IL-2, IL-4, IL-5, IL-6, IL-7, IL-8, IL-9, IL-10, IL-12, IL-13, IL-17A, IL-21, TNF-α, IFN-γ, CCL11 (also known as eotaxin), and CCL5 (also known as RANTES). For a full description of the methods, see this article’s Methods section in the Online Repository at www.jacionline.org. Of note, previous studies have shown that cytokine levels in the middle meatus mucus mirror those of olfactory cleft and of nasal cavity and polyp tissue samples, supporting the use of this method in this study.^13,24,25^

### Histopathologic evaluation of sinonasal tissue

Sinonasal tissue collected from the ethmoid bulla or the posterior ethmoid sinus during surgery underwent histopathologic evaluation with hematoxylin and eosin-staining by a pathologist. The mean number of eosinophils over 5 randomly selected HPFs was determined for each patient.

### Participant demographic characteristics and clinical factors

Patient age, sex, race, body mass index, medical history, and comorbid conditions including asthma, allergic rhinitis, aspirin-exacerbated respiratory disease (AERD), allergic fungal rhinosinusitis (AFRS), and previous surgical history were recorded. A Lund-MacKay score was assigned for each patient for whom a high-resolution computed tomography scan of the paranasal sinuses was performed no more than 3 months before surgery. Patient-reported symptom severity was measured preoperatively and postoperatively using the 22-item Sinonasal Outcome Test (SNOT-22). In general, postoperative therapy after ESS varied slightly between providers but typically included a brief course of oral steroids immediately after the procedure, daily use of nasal corticosteroid sprays or steroid irrigations, and nasal saline rinses.

Date of polyp recurrence was defined as the first postoperative clinic visit in which frank polyps were noted, grade 2 (or higher) polyps according to the Meltzer Clinical Scoring System were observed, or when grade 1 polyps or polypoid changes were noted and steroids were prescribed at the same visit. The date of first postoperative rescue oral steroid course for any indication (after the course received immediately after the procedure) was also recorded for each patient. The date of biologic therapy (dupilumab, omalizumab, mepolizumab, or tezepelumab) for any indication first prescribed or noted in the medical record, whichever was earlier, was also recorded. This outcome was primarily focused on the prescription of biologics, rather than their initiation, because this was felt to better reflect the disease trajectory than the initiation of biologics, which can vary on the basis of access and insurance provider.

### Outcomes

The primary outcome of the project was time-to-polyp recurrence. Secondary outcomes were time-to-first rescue oral steroid course, time-to-prescription of dupilumab therapy, and time-to-prescription of any biologic therapy.

### Statistical analysis

Principal-component analysis (PCA) followed by hierarchical cluster analysis was used to identify CRSwNP endotype clusters, as described in previous studies.^6,11^ This statistical method was chosen because it is a well-established method of identifying inflammatory endotypes in CRS.^2,3,6,8,11,12,26^ This method also offers an unstructured approach to creating endotype clusters on the basis of patient similarities without needing to specify the number of clusters in advance, ultimately allowing for uncovering of more meaningful and representative clusters while minimizing bias.^27^

The PCA with varimax rotation was conducted on the 17-cytokine inflammatory profile after square root transformation because of highly positively skewed distributions.^6,11^ Missing values were imputed with average scores using the multiple imputation chained equations procedure. Five summary factors with an eigenvalue greater than 1 were chosen and this solution accounted for 69% of the variance in the data (see Fig E1 and Table E1 in this article’s Online Repository at www.jacionline.org). Hierarchical cluster analysis was then performed using these 5 PCA factors, and the elbow method was used to identify the number of clusters.

The primary inflammatory endotype characterization for each cluster was determined by comparing the median levels and the standardized means of cytokines within each cluster with those of other clusters, with the terms “high” and “low” being used in a relative context. Cytokines were grouped on the basis of their general associations with established inflammatory endotypes, although we acknowledge that many are linked to multiple inflammatory pathways.^1,2,28–30^ They were grouped as follows: IFN-γ, TNF-α, IL-12, and IL-2 (type 1 inflammation); IL-4, IL-5, IL-9, IL-13, and CCL11 (type 2 inflammation); IL-17, IL-21, IL-7, and IL-10 (type 3 inflammation); and IL-1β, IL-6, and IL-8 (innate inflammation).

Participants’ demographic characteristics and clinical factors were then summarized by clusters. Differences among clusters were assessed with the Kruskal-Wallis test for continuous variables or the Pearson chi-square test for categorical variables. The outcomes of disease burden, which included time-to-polyp recurrence, time-to-oral steroid course, time-to-prescription of dupilumab therapy, and time-to-prescription of biologic therapy, were compared using a log-rank test, and survival was estimated using the Kaplan-Meier method. For time-to-event analyses, time 0 was the date of surgery and follow-up time was truncated at 5 years. There were 6 patients who were prescribed dupilumab before surgery and 13 patients who were prescribed biologics before surgery, and they were excluded from their respective survival analyses.

Cox regression models were used to calculate hazard ratios (HRs) and 95%CIs. A univariate model was used for the outcomes of polyp recurrence. Multivariable models were used for analysis of oral steroid course with adjustments for asthma and for analysis of dupilumab and biologic therapy with adjustments for both asthma and date of surgery to account for the portion of our cohort that underwent surgery before dupilumab approval in June 2019.^31,32^ Two-sided *P* values less than or equal to .05 were considered statistically significant. All analyses were conducted using R version 4.1 (R Core Team 2024, Vienna, Austria).

## RESULTS

### Patient characteristics

A total of 269 patients with CRSwNP were included in the study. Patients were predominantly male (61.0% [n = 164]) and White (83.6% [n = 225]), and the median age at time of surgery was 48 years (interquartile range [IQR], 35–59) (Table I). Comorbid asthma was present in 52.8% (n = 142) of patients and allergic rhinitis was present in 64.3% (n = 173). Forty-nine patients (18.2%) had AERD and 47 patients (17.5%) had AFRS. There was notable improvement in SNOT-22 scores 3 months postoperatively (median, 14 [IQR, 5–28]) compared with preoperative SNOT-22 scores (median, 43 [IQR, 29–60]).

### Identification and characterization of inflammatory endotypes

**FIG 1.**
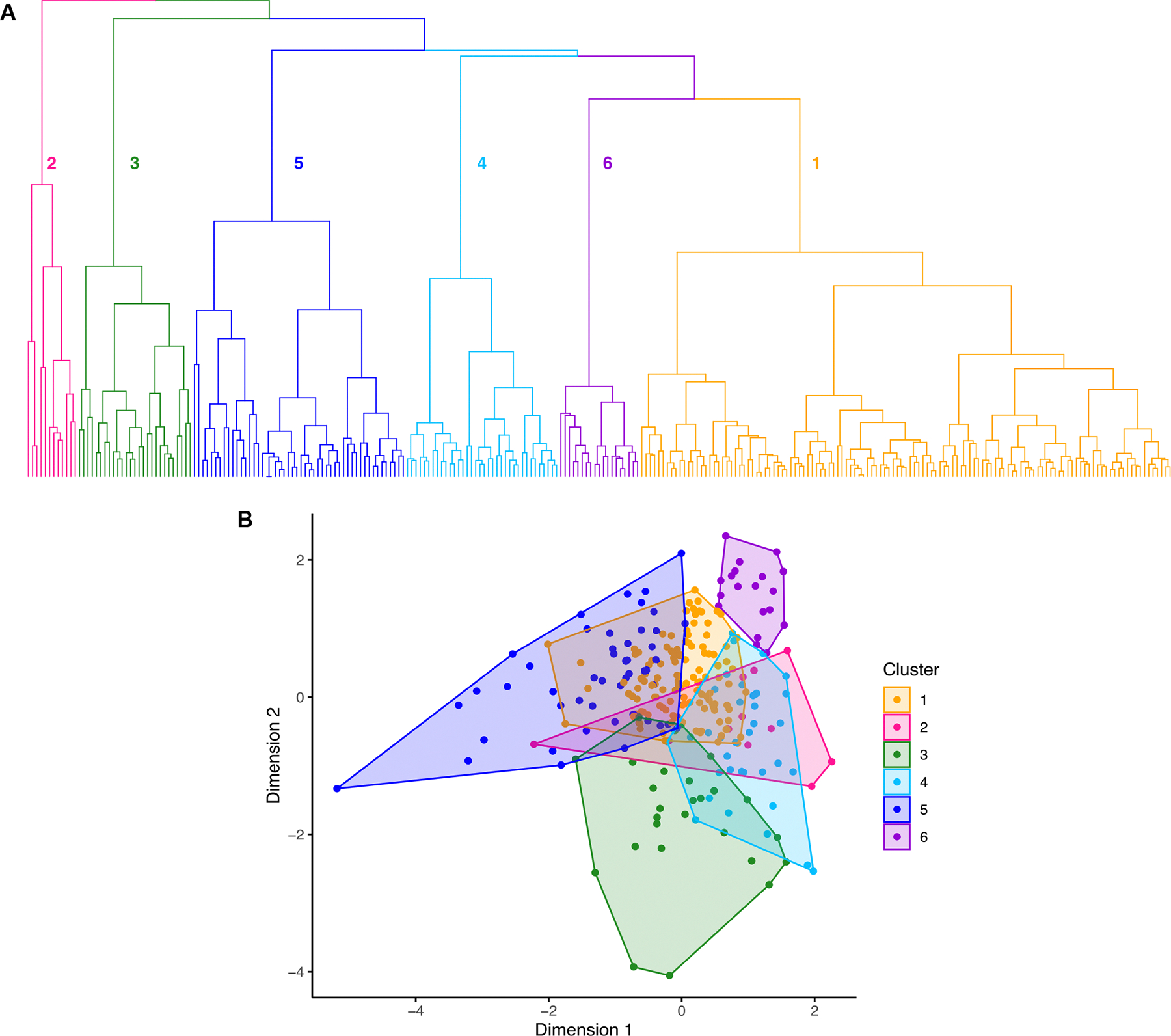
Identification of 6 inflammatory clusters in patients with CRSwNP using inflammatory cytokines. **A,** The dendrogram illustrates the results of the hierarchical cluster analysis of 17 inflammatory cytokines. Six distinct clusters were identified, representing groupings of patients with similar cytokine profiles. Each vertical line at the bottom of the dendrogram corresponds to an individual patient, and shorter horizontal branches between patients indicate greater similarity in cytokine profiles. **B,** The PCA plot depicts the distribution of patients within each of the 6 clusters and the relationships between these clusters. Each point represents an individual patient, and patients or clusters that are closer together exhibit more similar inflammatory profiles.

Using hierarchical cluster analysis, we identified 6 CRSwNP clusters on the basis of cytokine inflammatory profile (Fig 1). The patient demographic characteristics and clinical factors for each cluster are provided in Table I.

**FIG 2.**
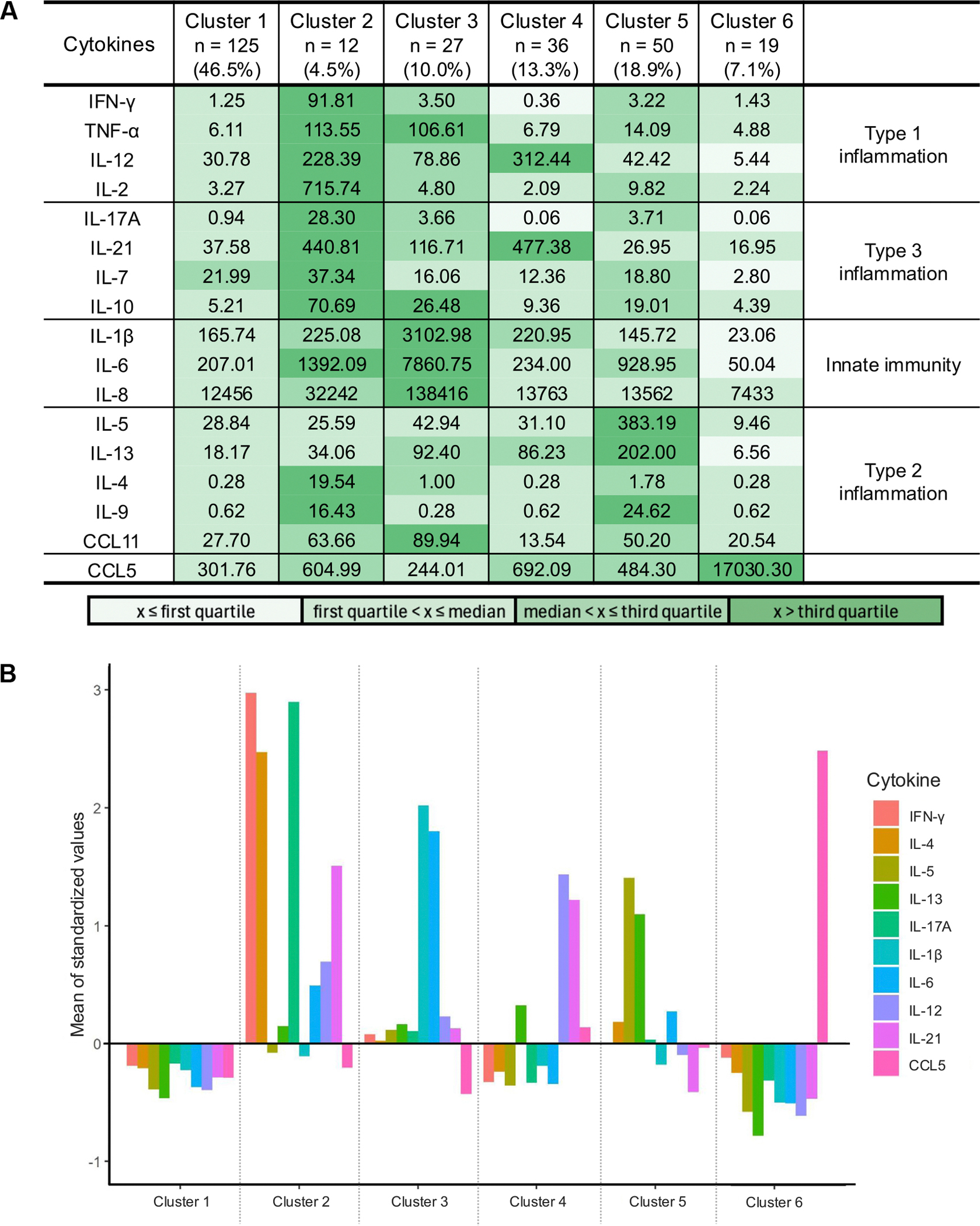
Cytokine characterization of CRSwNP clusters. **A,** Modified heatmaps of the median levels (pg/mL) of all 17 cytokines by cluster are shown. Cytokine levels are color coded by 4 shades of *green*, as indicated in the legend, on the basis of the median and first and third quartile values for each cytokine across all patients. **B,** The mean standardized levels of 10 cytokines (those most representative of the inflammatory endotypes) are shown for the clusters. Each bar represents a cytokine, with the height of the bar indicating how the mean cytokine level in each cluster compares with the overall cohort mean. Together, these show that cluster 1 exhibited relatively low levels of inflammatory cytokines, whereas cluster 2 was characterized by relative upregulation of type 1 (IFN-γ, TNF-α, IL-12, and IL-2) and type 3 (IL-17, IL-21, IL-7, and IL-10) inflammatory cytokines. Cluster 3 was defined by comparatively high levels of innate inflammatory cytokines (IL-1β, IL-6, and IL-8) and cluster 4 by relatively elevated levels of IL-12 and IL-21. Cluster 5 was distinguished by comparatively high levels of type 2 inflammatory cytokines (IL-4, IL-5, IL-9, IL-13, and CCL11), whereas cluster 6 was defined by relative upregulation of CCL5.

Each cluster exhibited a component of type 2 inflammation, characterized by elevated levels of IL-5 and IL-13 (Fig 2, A). They also demonstrated additional distinguishing cytokine characteristics on the basis of cytokine levels compared with other clusters. Cluster 1, totaling close to half of the study population (n = 125 [46.5%]), was characterized by relatively low levels of inflammatory cytokines, with nearly all median cytokine levels for this cluster falling below the overall median values (Fig 2). Meanwhile, cluster 2 (n = 12 [4.5%]) was defined by relatively elevated levels of IFN-γ, IL-4, and IL-17A, representing a predominantly mixed type 1 and type 3 inflammatory profile. When compared with other clusters, this group was significantly younger (median age, 35 years; IQR, 25–60; *P* = .026) and had a high prevalence of patients with AFRS (n = 4 [33%]; *P* <.001) (Table I). This cluster also had the lowest mean number of eosinophils per HPF, although this did not reach statistical significance (*P* = .072).

Cluster 3 (n = 27 [10.0%]) was characterized by comparatively high levels of IL-1β, IL-6, IL-8, and TNF-α, which is consistent with a predominantly innate, proinflammatory endotype (Fig 2). The patients in this group tended to be older (median age, 58 years; IQR, 43–66) than the patients in the remaining clusters (*P* = .026) (Table I).

Cluster 5 (n = 50 [18.9%]) was defined by relatively high levels of IL-5, IL-9, and IL-13, consistent with a fairly homogeneous type 2 inflammatory cluster (Fig 2). This group had the highest proportion of patients with AFRS (n = 18 [36%]; *P* < 001). It also had the highest mean number of eosinophils per HPF, although this was not statistically significant (*P* = .072) (Table I).

Both clusters 4 and 6, like cluster 1, showed relatively low levels of inflammatory cytokines (Fig 2). However, cluster 4 (n = 36 [13.3%]) was distinguished from clusters 1 and 6 by its relatively high levels of IL-12 and IL-21. Cluster 6 (n = 19 [7.1%]) was distinguished from clusters 1 and 4 by its significantly elevated levels of CCL5. Notably, there were no patients in cluster 6 with AFRS (*P* < .001) (Table I).

Patients in each cluster were otherwise similar in terms of sex, race, body mass index, asthma status, allergic rhinitis, comorbid AERD, and use of nasal corticosteroids and antileukotriene inhibitors (Table I). Perioperative factors such as Lund-MacKay computed tomography scores and a history of previous ESS were similar between groups. There were also no significant differences in preoperative SNOT-22 scores (*P* = .34) and postoperative SNOT-22 scores at 3 months (*P* = .52), 6 months (*P* = .085), 12 months (*P* = .73), 18 months (*P* = .88), and 24 months (*P* = .44) between clusters.

### Differences in disease trajectory between clusters

#### Polyp recurrence.

**FIG 3.**
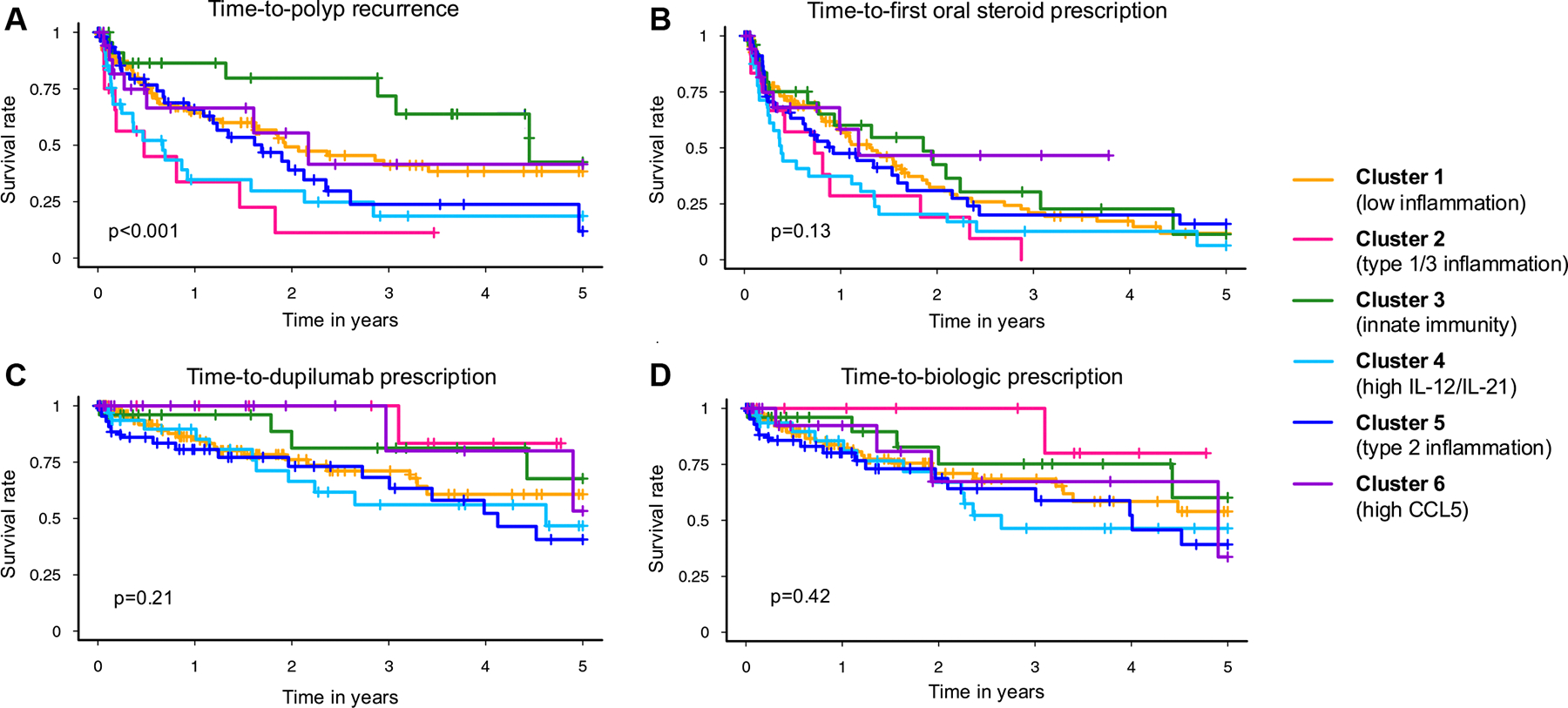
Kaplan-Meier curves for patients with CRSwNP by cluster. **A,** Time-to-polyp recurrence (in years) differed between clusters, with clusters 2 (mixed type 1 and type 3 inflammation) and 4 (IL-12^High^/IL-21^High^) exhibiting shorter time-to-polyp recurrence and cluster 3 (innate immunity) exhibiting longer time-to-polyp recurrence. **B-D,** Time-to-first oral steroid prescription (Fig 3, B), time-to-prescription of dupilumab (Fig 3, C), and time-to-prescription of biologic therapy (in years) (Fig 3, D) did not differ between clusters. Log-rank *P* values are shown.

**FIG 4.**
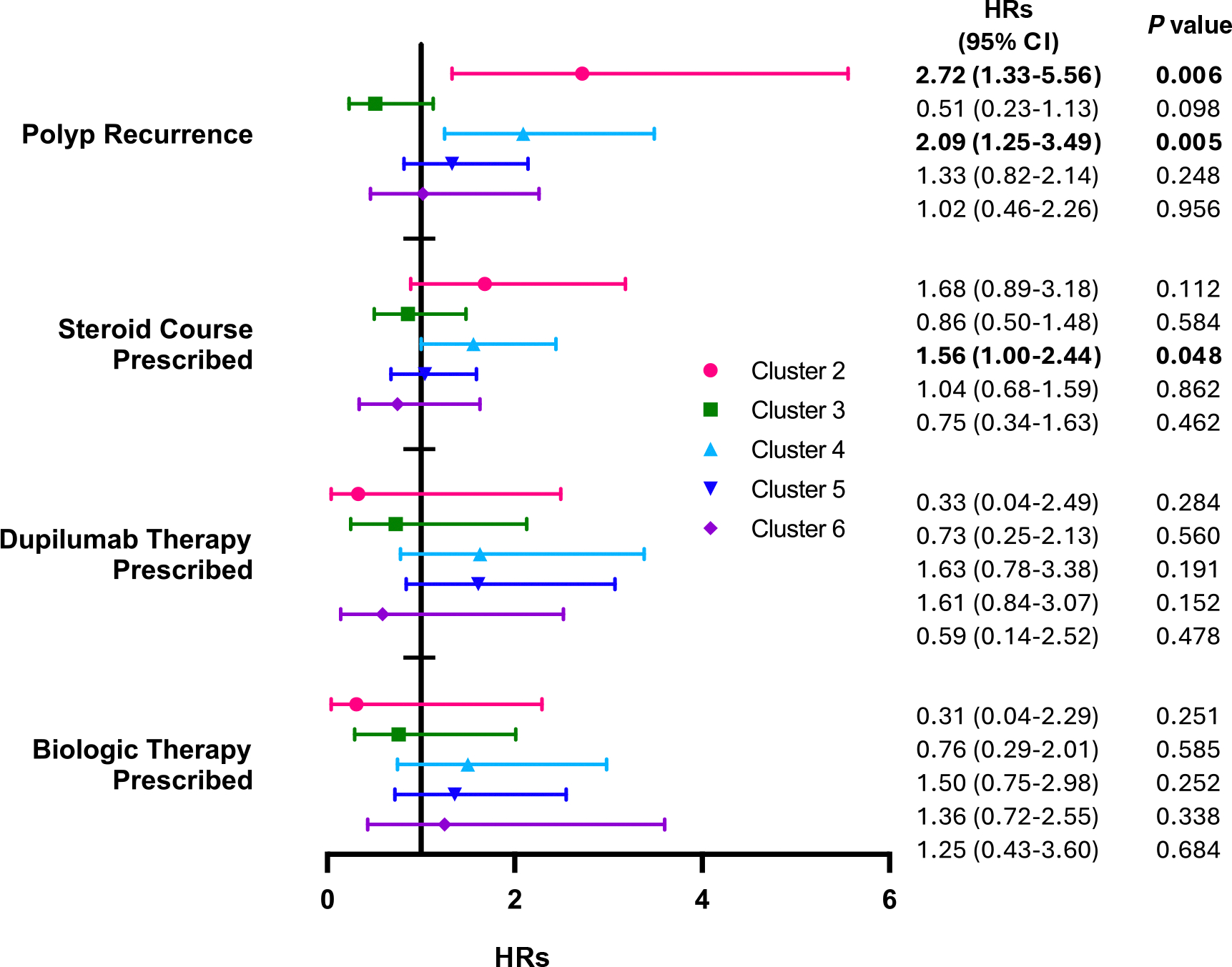
HRs for time-to-clinical outcome by CRSwNP inflammatory cluster. A forest plot is shown depicting unadjusted HRs and 95% CIs for time-to-polyp recurrence, as well as adjusted HRs for time-to-oral steroid course (adjusted for asthma) and time-to-prescription of dupilumab therapy and time-to-prescription of biologic therapy (both adjusted for asthma and date of surgery). The reference group was cluster 1 (low inflammation). HRs greater than 1 indicate an increased risk of the respective event, whereas values less than 1 suggest a reduced risk. Boldfaced values represent statistically significant associations (*P* < .05).

During the study period, 43.9% of patients (n = 118) experienced polyp recurrence, and the median time-to-polyp recurrence was 0.53 (IQR, 0.13–1.78) years (see Table E2 in this article’s Online Repository at www.jacionline.org). Overall, patients in clusters 2 (IFN-y^High^/IL-4^High^/IL-17A^High^) and cluster 4 (IL-12^High^/IL-21^High^) had the shortest time-to-polyp recurrence compared with all other clusters, whereas cluster 3 (IL-1β^High^/IL-6^High^/IL-8^High^) exhibited the longest time-to-polyp recurrence (*P* <.001; Fig 3, A). More specifically, when compared with cluster 1, patients in both cluster 2 (HR, 2.72; 95% CI, 1.33–5.56) and cluster 4 (HR, 2.09; 95% CI, 1.25–3.49) had significantly greater risk of polyp recurrence (Fig 4).

#### Oral steroid course.

Approximately 60% of patients (n = 163) received a dose of rescue oral steroids postoperatively (Table E2). The median time-to-oral steroid course was 0.50 (IQR, 0.15–1.53) years. Although time-to-steroid initiation was similar between clusters (*P* = .13; Fig 3, B), patients in cluster 4 were noted to have shorter time-to-oral steroids compared with those in cluster 1 (HR, 1.56; 95% CI, 1.00–2.44) when adjusting for asthma (Fig 4).

#### Biologic prescription.

Finally, 57 patients (21.7%) were prescribed dupilumab after surgery and 64 patients (25.0%) were prescribed a biologic after surgery, with a median time-to-prescription of 1.05 (IQR, 0.23–2.99) and 1.00 (IQR, 0.23–2.57) years, respectively (Table E2). Time-to-dupilumab prescription and time-to-biologic prescription were similar across clusters (*P* = .21 and *P* = .43, respectively) (Figs 3, C and D, and 4).

## DISCUSSION

Although demographic and phenotypic features have historically driven care pathways in CRS, more recent analyses have instead favored categorizing patients on the basis of inflammatory biomarkers that may more accurately reflect differences in disease pathobiology.^2,3,33,34^ In this study, we categorized patients with CRSwNP into 6 inflammatory endotypes using 17 mucus cytokines and investigated variations in disease trajectory between groups. To our knowledge, this is the first study to explore the relationship between CRSwNP inflammatory profiles and clinical outcomes in a Western population, and our findings have potential treatment implications for this otherwise phenotypically similar patient population.

### Disease trajectory of type 2 inflammatory endotype

In Western countries, type 2 eosinophilic inflammation has classically been the prevailing inflammatory profile associated with CRSwNP. Type 2 inflammation is traditionally characterized by elevated levels of IL-4, IL-5, IL-9, IL-13, IL-25, and IL-33 and with eosinophilia.^1,2,35^ Although our study was limited to patients with CRSwNP, a previous study by our group showed that IL-5 and IL-13 levels are significantly higher in patients with CRS (93.1 ± 286.5 and 67.5 ± 145.6 pg/mL, respectively) compared with control patients (0.5 ± 0.8 and 6.1 ± 11.6 pg/mL, respectively).^23^ In the present study, all clusters had median IL-5 and IL-13 levels above those of the controls noted in previous studies, suggesting that a component of type 2 inflammation was present in each cluster. Similarly, in their cluster analysis, Tomassen et al^2^ noted high IL-5 levels in 6 of 10 CRS clusters, with most of the patients in these clusters exhibiting a CRSwNP phenotype. These findings lend further support to the prominent role of type 2 inflammation in CRSwNP.

Although all disease clusters were characterized by some level of type 2 inflammatory burden, homogeneous type 2 inflammation was observed only in cluster 5. This cluster exhibited the highest levels of IL-5 and IL-13 and the largest number of tissue eosinophils on pathology, although this was not statistically different from other clusters. Cluster 5 also had the highest proportion of patients with AFRS, consistent with previous studies that have shown that type 2 inflammation plays a significant role in the pathogenesis of AFRS.^36,37^

Interestingly, time-to-oral steroid course and time-to-biologic prescription were similar between cluster 5 and other inflammatory clusters. Moreover, polyp-free survival in cluster 5 was similar to cluster 1 (low inflammation) and cluster 6 (CCL5^High^), both of which represented endotypes with low overall inflammatory burden. This suggests that although type 2 inflammation is a key player in the inflammatory pathway for CRSwNP, a purely type 2–predominant endotype does not necessarily portend worse outcomes.

These findings differ from a recent cluster analysis study by Wang et al^34^ that found that polyp recurrence was the greatest in a high type 2 inflammation cluster. Their analysis differed from ours in that they included both CRSsNP and CRSwNP. The inclusion of CRSsNP in the cluster analysis may have affected the patient distributions within each cluster, limiting generalizability of their findings to patients with CRSwNP alone. Meanwhile, our analysis benefited from focused analysis of phenotypically similar patients with CRSwNP and allowed us to identify nuanced outcome differences between clusters. Moreover, their patient population consisted of primarily Asian patients and they noted that those in the high type 2 inflammatory endotype had lower levels of IL-5 and IgE and higher levels of IL-6 and IL-8 than those in similar endotypes in the European population.^2,34^ Several other studies have also noted differences in CRSwNP endotypes between Asian and Western patients, which could further explain the differences between our studies’ findings.^35,38,39^

### Disease trajectory of mixed type 1 and type 3 inflammatory endotype

In contrast, we found that cluster 2 (IFN-γ^High^/IL-4^High^/IL-17A^High^) exhibited significantly reduced polyp-free survival compared with other clusters. This cluster was characterized by mixed type 1 and type 3 inflammation, a heterogeneous endotype that has previously been described.^2,3^ In addition, this cluster had the highest proportion of patients requiring oral corticosteroids during the follow-up period (Table E2), and although not statistically different from other clusters, it had the lowest mean tissue eosinophil count. Overall, these findings suggest that patients with coexisting non–type 2 inflammation have worse disease trajectory.

These findings are in line with previous research by Ryu et al,^40^ which noted that polyp recurrence requiring revision surgery was associated with elevated IFN-γ and IL-17 levels. Moreover, increased levels of IL-17 and IFN-γ have been shown to be associated with steroid-resistant asthma.^41^ This observation is presumably because corticosteroids preferentially diminish inflammatory pathways downstream of type 2 inflammatory mediators. We hypothesize that a similar mechanism is at play in patients with CRSwNP, with patients carrying substantial type 1 and/or type 3 inflammatory burden benefiting less from corticosteroids compared with other patients, which results in a greater likelihood of polyp recurrence.

Cluster 4 (IL-12^High^/IL-21^High^) was also associated with an increased likelihood of polyp recurrence compared with other clusters. Moreover, although there were no significant differences in survival curves for time-to-oral steroid course, cluster 4 was associated with a shorter time-to-oral steroid course compared with cluster 1 when adjusting for asthma in the Cox regression model (HR, 1.56; IQR, 1.00–2.44).

Interestingly, in addition to demonstrating the worst polyp-free survival, both cluster 2 (IFN-γ^High^/IL-4^High^/IL-17A^High^) and cluster 4 (IL-12^High^/IL-21^High^) exhibited the highest levels of IL-12 and IL-21 among the clusters (Fig 2), signaling the potential importance of IL-12 and IL-21 in the pathogenesis of polyp recurrence. IL-12 has been associated with T_H_1-cell differentiation, and IL-21 is linked with T_H_17-cell differentiation.^28,29^ Moreover, Xiao et al^42,43^ previously showed that IL-12 increases levels of IL-21 in polyp tissue, that IL-21 is frequently upregulated in nasal polyps, and that an elevated level of IL-21 is associated with polyp recurrence.

Our study findings highlight a common characteristic in endotypes that may be associated with poorer outcomes, namely, an abundance of inflammatory cytokines linked with mixed type 1 and type 3 inflammation and differentiation. This potential association warrants further investigation because biologics currently used in the treatment of CRSwNP all target type 2 inflammation.^44^ Our study suggests that therapeutic alternatives for CRSwNP that target type 1 and type 3 inflammation may be needed given the association of mixed inflammation with polyp recurrence in our study, although further studies investigating this relationship are needed.

### Disease trajectory of innate immunity endotype

Our results also identified a subgroup of patients (cluster 3, IL-1β^High^/IL-6^High^/IL-8^High^) that was characterized by predominantly proinflammatory, innate immunity cytokines. This mixed neutrophilic and type 2 inflammatory endotype is an increasingly recognized endotype in both Asian and Western countries.^2,3,45–47^ In our study, this cluster also represented the oldest cohort, which supports previous findings from our group showing that patients with CRS of advanced age are more likely to have neutrophilic inflammation and elevated cytokines associated with innate immune function.^11^

Interestingly, these patients were the least likely to demonstrate polyp recurrence over time. These findings somewhat contrast those from the study by Liao et al,^3^ which determined that patients with CRS with neutrophilic inflammatory endotype had high proportions of “difficult-to-treat” disease. Similar to the study by Wang et al,^34^ their cluster analysis included both CRSsNP and CRSwNP and primarily consisted of Asian patients, limiting comparison of their findings to patients with CRSwNP alone and to Western patients.

### Comparison of time-to-biologic prescription between endotypes

Finally, although there were notable differences in time-to-polyp recurrence and time-to-oral steroid course between clusters, our results revealed no significant differences between clusters in time-to-prescription of biologic therapy or time-to-prescription of dupilumab alone, even when controlling for asthma and date of surgery. These findings may be due to the relatively low number of patients in our study who were prescribed biologics (n = 64), the initiation of biologics for reasons other than nasal polyps, or subjective differences in clinical decision making, which were not accounted for in the present study. As biologics continue to grow in use, studies should continue to investigate differences in outcomes and in responses to biologics on the basis of inflammatory endotype because this could further refine management options for patients.

### Limitations

There are several limitations to this study. Patients were all from a single tertiary-care institution located in the southern United States. Several environmental, geographic, and immunopathologic differences have been established among patients with CRS, and thus our findings may not be generalizable to other care settings.^35,48^ An additional limitation is that our study population included only patients who underwent ESS and did not characterize patients who did not require surgery. Although this may bias toward patients with more severe disease, these are the patients with the greatest treatment needs, and a better understanding of their disease can lead to enhanced management strategies. Another limitation is that mucus and tissue samples were obtained at a single time point (intraoperatively) and thus cannot account for changes in inflammatory endotype that may occur over time or that may result from changes in environmental factors. Our group has previously shown that endotype assignment can change after surgical intervention, and further studies investigating these changes are needed.^49^

In addition, our time-to-polyp recurrence analysis did not account for the use of biologics, which could have contributed to prolonged recurrence times and posed a limitation to our findings. However, because biologics are typically initiated after polyp recurrence and biologic prescription rates were not significantly different between clusters, we suspect that the impact of the use of biologics was relatively minor. Finally, the study was limited by the small size of several of the clusters and the sole inclusion of inflammatory cytokines with exclusion of remodeling parameters, cellular contents, and clinical variables in the creation of the clusters. Nevertheless, our study identified clusters that resemble those described in previous studies, which helps validate this approach and our findings.^2,3^

## Conclusion

Our study provides further evidence on the complex, heterogeneous inflammatory profiles underlying CRSwNP and the possible implications for disease trajectory. Although most patients in our study had a low inflammatory burden and less than half of the patients experienced polyp recurrence, patients with a mixed type 1 and type 3 inflammatory endotype and/or elevated levels of IL-12 and IL-21, which are associated with type 1 and type 3 inflammation, had more rapid polyp recurrence after surgery. Meanwhile, patients with a purely type 2–dominant inflammatory endotype did not exhibit worse outcomes. Although additional studies are needed, our findings highlight key differences between CRSwNP endotypes and suggest that clinical outcomes differ by endotype. In the long-term, there may be a need for targeted treatments beyond the currently available type 2 inflammatory modulating biologics to address severe disease in select patients with CRSwNP.

## Supplementary Material

1

## Figures and Tables

**TABLE I.** Patient characteristics and clinical factors by cluster

Variable	All	Cluster 1	Cluster 2	Cluster 3	Cluster 4	Cluster 5	Cluster 6	*P* value

Total patients	269 (100.0)	125 (46.5)	12 (4.5)	27 (10.0)	36 (13.4)	50 (18.6)	19 (7.1)	
Age (y)	48 (35–59)	**50 (36–60)**	**35 (25–60)**	**58 (43–66)**	**45 (35–54)**	**40 (34–52)**	**49 (43–56)**	**.026**
Sex: female	105 (39.0)	48 (38.4)	3 (25.0)	12 (44.4)	15 (41.7)	21 (42.0)	6 (31.6)	.84
Race: White	225 (83.6)	97 (77.6)	11 (91.7)	23 (85.2)	33 (91.7)	44 (88.0)	17 (89.5)	.28
BMI (kg/m^2^)	28 (25–32)	28 (25–32)	31 (26–33)	29 (27–33)	27 (25–30)	28 (26–32)	29 (25–32)	.81
Asthma	142 (52.8)	62 (49.6)	7 (58.3)	13 (48.1)	20 (55.6)	32 (64.0)	8 (42.1)	.49
Allergic rhinitis	173 (64.3)	80 (64.0)	8 (66.7)	16 (59.3)	22 (61.1)	34 (68.0)	13 (68.4)	.97
AERD	49 (18.2)	19 (15.2)	1 (8.3)	5 (18.5)	6 (16.7)	15 (30.0)	3 (15.8)	.27
AFRS	47 (17.5)	**19 (15.2)**	**4 (33.3)**	**2 (7.4)**	**4 (11.1)**	**18 (36.0)**	**0 (0.0)**	**<.001**
Nasal corticosteroid user	210 (78.1)	97 (77.6)	10 (83.3)	21 (77.8)	27 (75.0)	39 (78.0)	16 (84.2)	.97
Antileukotriene inhibitor user (n = 219)	73 (33.3)	29 (27.9)	3 (30.0)	9 (37.5)	9 (28.1)	18 (50.0)	5 (38.5)	.25
Previous surgery (n = 220)	101 (45.9)	42 (40.4)	5 (50.0)	14 (56.0)	13 (40.6)	20 (55.6)	7 (53.8)	.49
CT score (n = 202)	17 (13–20)	16 (13–19)	20 (14–22)	18 (13–20)	16 (12–20)	20 (15–22)	16 (14–21)	.16
Mean eosinophils/HPF (n = 218)	91.0 (32.2–123.2)	94.0 (30.0–116.9)	31.5 (3.2–114.5)	54.0 (20.0–100.0)	96.0 (45.0–122.8)	100.0 (73.8–167.8)	61.0 (15.0–100.0)	.072
SNOT-22 scores								
Preoperative (n = 181)	43 (29–60)	42 (28–61)	37 (23–56)	50 (34–62)	38 (27–53)	50 (29–61)	59 (39–74)	.34
3 mo postop (n = 102)	14 (5–28)	14.5 (5–36)	23 (14–32)	16.5 (8–21)	17.5 (12.8–23.5)	9 (0.8–20.8)	24 (11–31.8)	.52
6 mo postop (n = 64)	16.5 (5.8–32.2)	19.5 (5–27.8)	14 (11–38.5)	23 (7–30)	16.5 (8.5–37.2)	6 (4–14)	33 (26–70)	.085
12 mo postop (n = 60)	14 (6.8–30.5)	10 (5.5–29.2)	25 (16–30)	23 (16.2–39)	19 (10.2–29.2)	13 (5–41)	26.5 (21.8–33.8)	.73
18 mo postop (n = 31)	20 (11.5–46)	17 (7–40)	19 (19–19)	37 (25–55)	23 (20–37)	22 (3.5–47.5)	23.5 (22.8–24.2)	.88
24 mo postop (n = 23)	22 (18–27)	21 (13.5–23)	44 (35–53)	20.5 (19.8–21.2)	25 (20.8–30.2)	23.5 (7.2–53.2)	10.5 (6.2–14.8)	.44

Data are presented as n (%) or median (IQR). Boldfaced values indicate *P* < .05.

*BMI*, Body mass index; *CT*, computed tomography.

## Key messages

- Several translational research studies have characterized distinct and clinically relevant inflammatory CRS endotypes, but few have investigated how disease trajectory and clinical outcomes differ between CRSwNP endotypes.
- Patients with CRSwNP with a mixed type 1 and type 3 inflammatory endotype or elevated levels of IL-12 and IL-21 (associated with type 1 and type 3 inflammation) have more rapid polyp recurrence, whereas patients with a type 2–dominant inflammatory endotype do not exhibit worse outcomes.
- These findings emphasize the heterogeneous nature of CRSwNP and suggest key differences in clinical outcomes between CRSwNP endotypes.

## Abbreviations used

AERD: Aspirin-exacerbated respiratory disease
AFRS: Allergic fungal rhinosinusitis
CRSsNP: Chronic rhinosinusitis without nasal polyps
CRSwNP: Chronic rhinosinusitis with nasal polyps
ESS: Endoscopic sinus surgery
HR: Hazard ratio
IQR: Interquartile range
PCA: Principal-component analysis
SNOT-22: 22-item Sinonasal Outcome Test
