# Supplementary material for: Effects of inflammatory endotypes on disease trajectory in chronic rhinosinusitis with nasal polyps
Source: J Allergy Clin Immunol. Author manuscript; Available in PMC 2026 Jun 29. (PMC13310653; doi:10.1016/j.jaci.2025.03.029)
Supplement: 1 [file NIHMS2174422-supplement-1.pdf]

METHODS

Mucus cytokine collection and analysis

Mucus was collected and processed using the following method. At the beginning of each surgery, 2 polyurethane sponges (9 × 24 mm) (Summit Medical, St Paul, Minn) were placed bilaterally into the middle meatus under endoscopic guidance. After 5 minutes, each sponge was removed and placed in a sterile microcentrifuge tube for immediate processing. The sponges were then placed into a microporous centrifugal filter device (MilliporeSigma, Billerica, Mass) and centrifuged at 14,000*g* for 10 minutes before being gently vortexed and then centrifuged for an additional 5 minutes to remove cellular debris. Supernatants were then removed, placed into a new microcentrifuge tube, and frozen at −80°C for subsequent analysis.

Mucus samples were analyzed and quantified using the following method. Multiplex cytokine assays were performed using a multiplex cytokine bead assay (BD Biosciences, Franklin Lakes, NJ) according to the manufacturer's protocol. In brief, 50 μL of mucus was mixed with 50 μL of mixed capture beads for each measured inflammatory mediator and incubated for 1 hour. Fifty microliters of mixed detection reagent was then added to each sample and standard before incubating for another 2 hours. Subsequently, 1 mL of wash buffer was added to the samples. The samples were centrifuged at 200*g* for 5 minutes and the supernatant was discarded. The beads were then resuspended in 300 μL of wash buffer and analyzed on a flow cytometer (LSR Fortessa flow cytometer, BD Biosciences, San Jose, Calif). Data were analyzed using the BD FCAP Array Software version 3.0 (BD Biosciences).

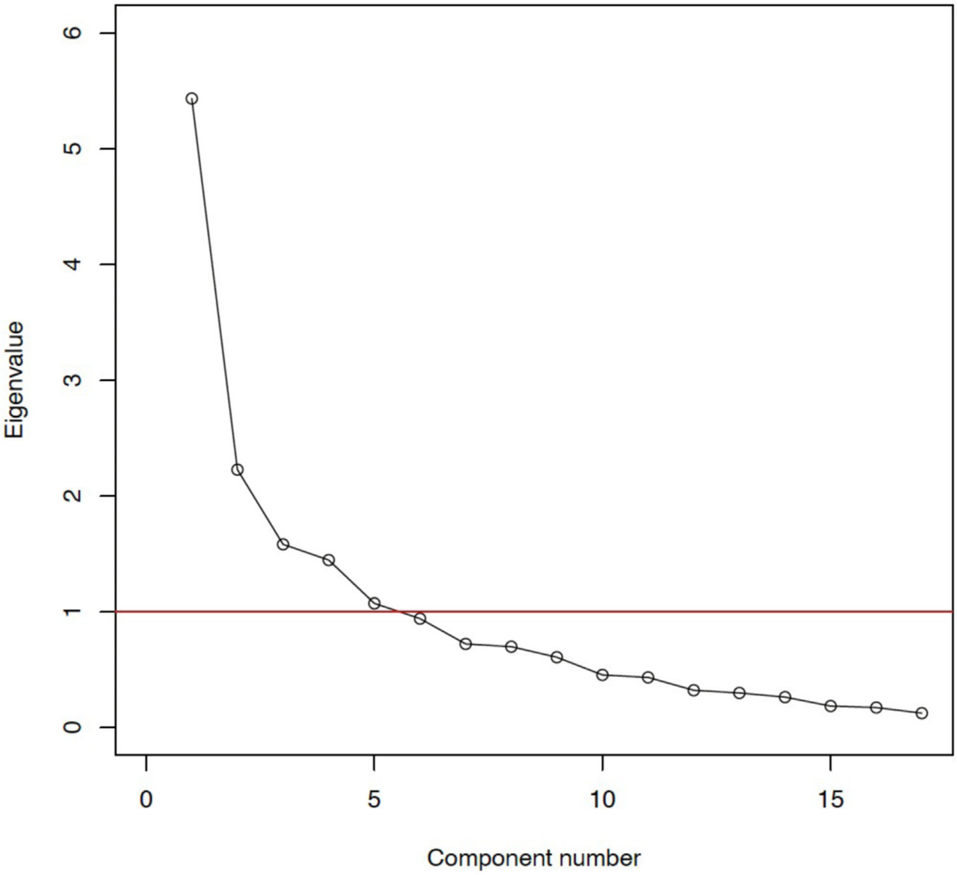

FIG E1. Validating the number of principal components. This Scree plot shows eigenvalues for the calculated factors. The optimal number of factors was estimated by identyfing factors with an eigenvalue greater than 1 and an approximate break point on the curve.

TABLE E1. Cytokine factor loading from PCA with varimax rotation

Variable	Rotated factor 1	Rotated factor 2	Rotated factor 3	Rotated factor 4	Rotated factor 5
IFN-γ	0.863				
IL-17A	0.857				
IL-4	0.811				
TNF-α	0.683				
IL-2	0.593				
IL-10	0.526				
IL-1β		0.874			
IL-6		0.767			
IL-8		0.766			
CCL11		0.458			
IL-5			0.905		
IL-13			0.851		
IL-9			0.636		
IL-12				0.860	
IL-21				0.816	
IL-7					0.602
CCL5					−0.799

TABLE E2. Frequency and time-to-outcomes by cluster

Variable	All	Cluster 1	Cluster 2	Cluster 3	Cluster 4	Cluster 5	Cluster 6
Patients, n	269	125	12	27	36	50	19
Polyp recurrence noted, n (%)	118 (43.9)	48 (38.4)	9 (75.0)	7 (25.9)	21 (58.3)	26 (52.0)	7 (36.8)
Median time-to-polyp recurrence (y) (IQR)	0.53 (0.13-1.78)	0.55 (0.13-1.66)	0.29 (0.1-0.97)	1.21 (0.21-3.41)	0.23 (0.11-0.88)	0.8 (0.23-1.76)	0.48 (0.13-1.77)
Received oral steroid course, n (%)	163 (60.6)	71 (56.8)	11 (91.7)	16 (59.3)	27 (75.0)	31 (62.0)	7 (36.8)
Median time-to-oral steroid course (y) (IQR)	0.50 (0.15-1.53)	0.53 (0.15-1.55)	0.57 (0.23-1.12)	0.77 (0.2-2.02)	0.28 (0.12-1.24)	0.55 (0.2-1.37)	0.34 (0.13-1.09)
Prescribed dupilumab therapy, n (%)	57 (21.7)	23 (18.7)	1 (8.3)	4 (15.4)	11 (30.6)	16 (33.3)	2 (11.1)
Median time-to-dupilumab prescription (y) (IQR)	1.05 (0.23-2.99)	0.86 (0.24-2.16)	2.96 (0.88-3.62)	1.68 (0.38-4.23)	1.26 (0.15-3.11)	1.13 (0.23-3.16)	1.26 (0.21-2.84)
Prescribed biologic therapy, n (%)	64 (25.0)	26 (22.0)	1 (9.1)	5 (19.2)	12 (33.3)	16 (34.0)	4 (22.2)
Median time-to-biologic prescription (y) (IQR)	1.00 (0.23-2.57)	0.84 (0.23-2.13)	2.82 (0.72-3.44)	1.39 (0.38-3.57)	1.08 (0.15-2.44)	1.10 (0.23-2.7)	0.87 (0.2-1.93)
